# Reconstructive and Regenerative Therapy of Atrophic Jaws with New Implant Techniques: Preclinical and Clinical Studies

**DOI:** 10.1155/2017/8252383

**Published:** 2017-03-21

**Authors:** Carmen Mortellaro, Sérgio Alexandre Gehrke, Eitan Mijiritsky

**Affiliations:** ^1^Amedeo Avogadro University of Eastern Piedmont, Novara, Italy; ^2^Bioface Institut, Santa Maria, RS, Brazil; ^3^Catedra de Biotecnologia, Universidad Católica de Murcia (UCAM), Murcia, Spain; ^4^Universidad Católica del Uruguay, Montevideo, Uruguay; ^5^Biotecnos-Tecnologia e Ciencia Ltda, Santa Maria, RS, Brazil; ^6^Tel Aviv University, Tel Aviv, Israel

Nowadays, dental implants represent a reliable and successful procedure for the prosthetic restoration of partially and totally edentulous patients, with high survival and success rates in the short, medium, and long term.

However, a sufficient amount of bone—in terms of height and width of the residual bone crest—is needed to place dental implants in the proper position and inclination, to allow the placement of a biologically, functionally, and aesthetically integrated prosthetic restoration.

Therefore, in the last years, several bone regeneration techniques have been developed, to allow proper placement of dental implants and the successful prosthetic restorations of patients with different types of bone defects. Among these techniques, there are onlay/inlay block regeneration, guided bone regeneration (GBR) with membranes, split crest techniques, maxillary sinus augmentation, and many others.

The objective of the bone regeneration is to promote the formation of new bone, in order to reconstruct an atrophic alveolar ridge before or in conjunction with implant placement, through the use of different biomaterials (autografts, allografts, xenografts, and synthetic materials) alone or in conjunction with biostimulants (growth factors or stem cells).

At the moment, in the international scientific community, there is great interest in the surgical techniques used for bone regeneration, from the most conventional (x example, bone regeneration with autografts or allografts blocks, or GBR with membranes) to the most modern and revolutionary (such as regeneration with custom-made synthetic scaffolds obtained with the modern digital technologies).

Moreover, bone regeneration is related to technological development and the discovery of new materials. For example, the biological regeneration with platelet concentrates is today another important interdisciplinary field of research, in which engineering principles and basic sciences are used to develop biological substitutes that can repair and regenerate the function of bone tissue damaged by trauma, degenerative diseases.

Today, some progress has been made in specific surgical applications, such as GBR and maxillary sinus augmentation, that represent safe and predictable treatment procedures; the real “last challenge” of biomaterials research in dentistry appears to be the vertical bone regeneration through onlay blocks of different materials, supported by a valid blood perfusion that can guarantee that “biological push” that eases the regeneration process within the entire block.

In the present special issue you will find a collection of articles dealing with different strategies for the bone regeneration in dentistry and maxillofacial surgery.

*Carmen Mortellaro* *Sérgio Alexandre Gehrke* *Eitan Mijiritsky*

